# Association of Carbonic Anhydrase 9 Polymorphism and the Epithelial Growth Factor Receptor Mutations in Lung Adenocarcinoma Patients

**DOI:** 10.3390/diagnostics10050266

**Published:** 2020-04-29

**Authors:** Ya-Yen Yu, Hui-Ling Chiou, Shih-Ming Tsao, Chen-Cheng Huang, Chih-Yun Lin, Chia-Yi Lee, Thomas Chang-Yao Tsao, Shun-Fa Yang, Yi-Wen Huang

**Affiliations:** 1 Institute of Medicine, Chung Shan Medical University, Taichung 402, Taiwan; yy68642@gmail.com; 2 Department of Clinical Laboratory, Changhua Hospital, Changhua 513, Taiwan; 3 School of Medical Laboratory and Biotechnology, Chung Shan Medical University, Taichung 402, Taiwan; hlchiou@csmu.edu.tw; 4 Department of Clinical Laboratory, Chung Shan Medical University Hospital, Taichung 402, Taiwan; 5 Division of Chest, Department of Internal Medicine, Chung Shan Medical University Hospital, Taichung 402, Taiwan; tsmhwy@ms24.hinet.net (S.-M.T.); his885889@gmail.com (T.C.-Y.T.); 6 School of Medicine, Chung Shan Medical University, Taichung 402, Taiwan; 7 Institute of Biochemistry, Microbiology and Immunology, Chung Shan Medical University, Taichung 402, Taiwan; 8 Division of Chest Medicine, Department of Internal Medicine, Taichung Hospital, Ministry of Health and Welfare, Taichung 403, Taiwan; jerryhuang123@gmail.com; 9 Department of Health, Pulmonary and Critical Care Unit, Changhua Hospital, Changhua 500, Taiwan; lin.chihyun@gmail.com; 10 Department of Ophthalmology, Show Chwan Memorial Hospital, Changhua 500, Taiwan; ao6u.3msn@hotmail.com; 11 Department of Medical Research, Chung Shan Medical University Hospital, Taichung 402, Taiwan

**Keywords:** carbonic anhydrase 9, epidermal growth factor receptor, single nucleotide polymorphism, lung adenocarcinoma, tumor stage, lymph node

## Abstract

Carbonic anhydrase 9 (CA9) plays a vital role in lung cancer progression. The current study explored the effect of CA9 gene polymorphisms and the epidermal growth factor receptor (EGFR) mutations on the clinicopathological characters of lung adenocarcinoma. In this study, three loci of CA9 single nucleotide polymorphism (SNP) (rs2071676 A > G, rs3829078 A > G, and rs1048638 C > A) were genotyped using the TaqMan allelic discrimination method in 193 EGFR wild type individuals and 281 EGFR mutation subjects. After adjusting for age, gender, and cigarette smoking status in logistic regression, all three CA9 SNPs illustrated a non-significant difference for the distribution between the EGFR wild type group and EGFR mutation group. Nevertheless, a significantly lower rate of CA9 SNP rs2071676 AG (adjusted odds ratio (AOR): 0.40, 95% confidence interval (CI): 0.16–0.95, *p* = 0.039) and AG + GG (AOR: 0.43, 95% CI: 0.18–0.98, *p* = 0.046) were found in the male population with L858R EGFR mutation compared to men with EGFR wild type. In addition, the CA9 SNP rs2071676 AG + GG genotype were significantly correlated to the lower tumor stage of lung adenocarcinoma in the whole study population (*p* = 0.044) and EGFR wild type individuals (*p* = 0.033). For the male population, the presence of CA9 SNP rs2071676 AG + GG genotype was also correlated to a lower tumor stage (*p* = 0.037) and fewer lymph node invasion (*p* = 0.003) in those with EGFR wild type. In conclusion, the existence of CA9 SNP rs2071676 is associated with the rate of EGFR L858R mutation in males. Furthermore, the CA9 SNP rs2071676 is correlated to lower tumor stage and lower risk for developing lymph node metastasis in lung adenocarcinoma, mainly in the EGFR wild type.

## 1. Introduction

Lung cancer is the leading etiology of cancer death in both males and females with a 1.6 million deaths worldwide in 2013 [1]. The lung adenocarcinoma accounts for about 30%–50% of all lung cancers and is predominant in the male population [2]. In the Eastern Asian population, the incidence lung cancers was 190.63 per 100,000 person-years in China [3], while a numerically lower rate of lung cancers was found in Taiwan with the incidence of 49.86 per 100,000 person-years [4]. For the distribution of different lung cancer subtypes, lung adenocarcinoma accounts for about 30%–50% percent of all the lung cancers worldwide (including Japan and Hong Kong) and is predominant in the male population [2]. In Taiwan, the ratio of lung adenocarcinoma in all lung cancers was nearly 54% in previous research [4,5], and a lower smoking rate, earlier clinical T-status, as well as greater metastasis tendency was observed in lung adenocarcinoma compared to lung squamous cell carcinoma in Taiwanese [5]. Except conventional treatments of lung adenocarcinoma, including radiotherapy and chemotherapy [6], the molecular target therapy that focuses on genetic aberrations of tumor cells was introduced recently and yielded favorable outcomes in lung adenocarcinoma [7]. In a previous study, the adjuvant of target therapy can elevate the longer progression-free survival and overall survival compared to the traditional management only [8].

The alteration of epidermal growth factor receptor (EGFR) is a prominent feature in the development of lung adenocarcinoma and its mutation is an available site for target therapy [9]. In the Asian population, the ratio of EGFR mutations in lung adenocarcinoma was about 40%–60%, which including the common L858R, Exon 19 in-frame deletion and T790M [10]. Additionally, young patients had a significantly higher ratio of EGFR mutations than the older population in Taiwan [11]. There are several forms of EGFR mutations that show the potentiality to be therapeutic or prognostic factors [12,13,14]. The L858R expression is a prevalent type of EGFR mutation that could harbor to the micropapillary component of lung adenocarcinoma and elevate the probability of tumor recurrence [15]. On the other hand, the Exon 19 in-frame deletion is another common EGFR mutation that revealed a better disease-free survival and overall survival of lung adenocarcinoma compared to those with the L858R mutation [16,17]. In addition, the co-existence of EGFR mutations and other genetic polymorphism that influence the prognosis of lung adenocarcinoma is not uncommon; the co-existence of EGFR mutation and endothelial nitric oxide synthase would significantly aggravate the lymph node invasion of lung adenocarcinoma [18].

The carbonic anhydrase 9 (CA9) is a zinc-containing glycoprotein protein that regulates cell proliferation and serves as a dominant factor in the disease course of several types of neoplasm [19]. Previous studies have revealed that CA9 expression is associated with poor clinical outcomes in various cancers [20,21,22,23,24,25]. Moreover, the presence of CA9 single nucleotide polymorphism (SNP) was associated with the development of uterine cancer, urothelial cell carcinoma, colorectal carcinoma hepatocellular carcinoma, and oral cancer [19,26,27,28,29]. Hua et al., reports that the presence of CA9 3′untranslated region (UTR) SNP lead to advanced stages, larger tumor sizes, and shorter survival in those with hepatocellular carcinoma [28]. Moreover, a higher susceptibility to oral cancer was also found in patients with several CA9 SNPs and environment risk factors of betel chewing or tobacco consumption [29]. Nevertheless, there are very few studies that have evaluated the association between CA9 SNP and the course of lung adenocarcinoma. Moreover, since EGFR mutations are associated to other types of genetic polymorphism, some correlations between CA9 SNP and EGFR mutations that affect the presentation of lung adenocarcinoma may exist; so far, such research is absent.

Herein, we aim to investigate the correlation between certain CA9 SNPs and EGFR variation in patients with lung adenocarcinoma. In addition, the potential effects of different forms of CA9 SNP and EGFR mutations on the severity of lung adenocarcinoma were also evaluated.

## 2. Materials and Methods

### 2.1. Study Subjects and Ethics Statement

We recruited 474 lung adenocarcinoma patients from 2015 to 2019 at the Division of Chest Medicine of Cheng Ching Hospital, Taichung Hospital, Chung Shan Medical University Hospital in Taichung, and Changhua Hospital in Changhua, Taiwan. Subjects that were diagnosed with lung adenocarcinoma and had a follow-up period longer than six months in hospital were enrolled as the study population. A total of 474 patients with lung adenocarcinoma were included in the current study after the selection process. The demographic data including age, gender, and cigarette smoking status were collected from the medical document of all hospitals. In addition, the Tumor, Node, Metastasis (TNM) status and tumor stages were staged by two pulmonologists, while the three degrees of cell differentiation (including well differentiated, moderately differentiated, and poorly differentiated) were defined according to the American Joint Committee on Cancer manual. The current study was approved by the Institutional Review Board of the Chung Shan Medical University Hospital (CS17103; 18 January 2018). For the analysis of genetic polymorphism of the CA9, venous blood drawing was performed for the study population. After that, the blood sample was stored in an ethylenediaminetetraacetic acid-containing tube, then instantly centrifuged and preserved in a laboratory refrigerator at −80 °C for further analysis.

### 2.2. Genomic DNA Extraction and EGFR Sequencing

The DNA extraction and sequencing procedures of EGFR mutations were performed using previously described methods [18]. After the DNA genome extraction from the frozen lung tumor specimen, the types of EGFR including the wild-type and mutation variants were amplified via the polymerase chain reaction (PCR), and finally subjected to a DNA sequencing reaction.

### 2.3. The Genotyping of CA9 SNPs Via Real-Time PCR

Two SNPs in exons, including the rs2071676 (+201, G/A) in exon 1 and the rs3829078 (+1081, A/G) in exon 7, and one SNP in 3′-UTR of exon 11, the rs1048638 (+1584, C/A), were selected due to their dominant effects in other neoplasms [19,26,27,28]. The genotyping procedure of CA9 SNPs was also used in a manner that has already been established [19]. Firstly, the DNA was extracted from the leukocytes of the venous blood sample using QIAamp DNA kits (Qiagen, Valencia, Valencia, CA, USA) according to the manufacture’s instruction. Then the allelic discrimination of the selected three CA9 SNPs involving rs2071676 (G/A), rs3829078 (A/G), and rs1048638 (C/A) was evaluated via the usage of a ABI StepOne Real-Time PCR System (Applied Biosystems, Foster City, CA, USA). The results of real-time PCR were analyzed by SDS version 3.0 software (Applied Biosystems, Foster City, CA, USA).

### 2.4. Statistical Analyses

The SAS version 9.4 (SAS Institute Inc, NC, USA) was utilized for all the statistical analyses. Descriptive analysis was used to present the demographic data, tumor stage, and tumor cell differentiation between the EGFR wild type and EGFR mutation forms. A chi-square test was applied to analyze the demographics and clinical characteristics between the two groups, except that the age was analyzed via an independent *t*-test. The multiple logistic regression, which adjusted the age, gender, and tobacco consumption was used to calculate the adjusted odds ratios (AOR) with their 95% confidence intervals (CI) for the different CA9 SNPs distributions among the wild type EGFR, whole EGFR mutation population, the EGFR mutation with L858 expression, and the EGFR mutation with Exon 19 in-frame deletion. In the next step, the multiple logistic regression was applied again to evaluate the association between the clinicopathologic characteristics and each phenotype of EGFR with different CA9 SNP rs2071676 phenotypes. A *p*-value of 0.05 or less was regarded as a statistically significant difference via the application of two-tails probability at 95% CI.

## 3. Results

### 3.1. Demographic and Clinical Characteristics of Patients with Lung Adenocarcinoma

In the whole study population with 474 participants, 193 patients belonged to the EGFR wild type group while another 281 individuals presented as the EGFR mutation group. The mean age was similar between the two groups (*p* = 0.689), while the female predominant (*p* < 0.001) and lower frequency of cigarette smoking (*p* < 0.001) were found in the EGFR mutation group. The clinicopathologic characteristics of lung adenocarcinoma were grossly similar between the two groups regarding the stage (*p* = 0.689), tumor T-stage (*p* = 0.343), lymph nodes status (*p* = 0.999), and distal metastasis (*p* = 0.356). However, the ratio of cell differentiation was higher in the EGFR mutation group (*p* < 0.001). The details of demographic characteristics are shown in Table 1.

### 3.2. Associations between CA9 Genotypes and EGFR Mutations in Adenocarcinoma Patients

To survey whether a correlation exist between CA9 SNPs and different types of EGFR phenotype, we analyzed the distribution of each CA9 SNPs in different EGFR presentations including the wild type and the mutation types. After the multiple logistic regression analysis that considered age, gender, and cigarette smoking status, there was no significant difference concerning the distribution of all the three CA9 SNPs (rs2071676, rs3829078, and rs1048638) among EGFR wild type individuals as well as different EGFR mutations including L858R expression and Exon 19 in-frame deletion (all *p* > 0.05; Table 2). However, the AOR of rs2071676 AG (AOR: 0.40, 95% CI: 0.16–0.95, *p* = 0.039) and AG + GG (AOR: 0.43, 95% CI: 0.18–0.98, *p* = 0.046) were significantly lower in the male population with a L858R EGFR mutation compared to the male patients with EGFR wild type, while the distribution between CA9 SNPs and other EGFR presentation remained similar (Table 3).

### 3.3. Correlations between Polymorphic Genotypes of CA9 and the Clinical Status of Lung Adenocarcinoma Patients with EGFR Mutations

We further analyzed the potential correlation between the clinicopathologic characteristics of lung adenocarcinoma and the distribution of EGFR phenotype and CA9 SNP rs2071676. In the whole study population, the presence of CA9 SNP rs2071676 AG + GG were significantly correlated with a lower tumor stage of lung adenocarcinoma in both the whole study population (*p* = 0.044) and the EGFR wild type individuals (*p* = 0.033). Also, the risk of lymph node metastasis of lung adenocarcinoma was lower likely with the CA9 SNP rs2071676 AG + GG in the EGFR wild type group (*p* = 0.005). The tumor T-status, rate of distant metastasis and the distribution of cell differentiation of lung adenocarcinoma did not alter in different EGFR presentation with CA9 SNP rs2071676 (all *p* > 0.05; Table 4). In the subgroup analysis of the male population, the presence of CA9 SNP rs2071676 AG + GG was also correlated with an earlier tumor stage (*p* = 0.037) and less possibility of lymph node invasion (*p* = 0.003) of lung adenocarcinoma in those with EGFR wild type. There were still no difference concerning the tumor T status, rate of distant metastasis and the distribution of cell differentiation of lung adenocarcinoma in different EGFR presentation with different CA9 SNP rs2071676 (all *p* > 0.05; Table 5).

## 4. Discussion

In the current study, the prevalence of CA9 SNP rs2071676 AG and AG + GG was significantly lower in male patients with lung adenocarcinoma and the EGFR mutation of L858R expression. Moreover, the CA9 SNP rs2071676 AG + GG was correlated to a lower tumor stage and lower risk for developing lymph node metastasis in lung carcinoma accompanied by wide type of EGFR. On the contrary, the tumor T-status, rate of distant metastasis, and the distribution of cell differentiation did not alter by the polymorphism of CA9.

The glycoprotein CA9 has been studied in the recent years, whereby the expression was higher in wide spectrum of malignancy and showed the potential for cancer therapy [30,31,32]. Served as the mediator of intracellular pH that seldom present in the normal human cells, this zinc-containing enzyme may maintain the tumor proliferation in a hypoxic microenvironment and contribute to the tumor invasiveness as well as therapeutic resistance according to previous studies [19,33,34,35]. Concerning the specific cancer that is associated with the existence of CA9, the expression of CA9 was more common in prostate cancer than in nodular prostate hyperplasia [36], and the higher expression of CA9 was correlated to poor survival in certain types of breast cancers [37]. In addition to the CA9 itself, the SNP of CA9 also influence the disease course of malignancy to a large extends [26,28,29]. On the other hand, the EGFR and its mutations influence the progression and managements in various malignancies including the lung carcinoma [38,39,40]. In the research previously published, the presence of EGFR mutations, such as deletions in exon 19 and L858R mutation, led to a higher sensitivity to tyrosine kinase inhibitors in patients with non-small-cell lung cancer, including lung adenocarcinoma [41,42,43], while the T790M mutations of EGFR before treatment is related to a significantly shorter progression-free survival [41]. In addition, the reactions to hypoxic tumor environment between EGFR and CA9 are contrary [44], which may contribute to the tumor progression. Consequently, the distribution of CA9 SNP and EGFR phenotypes is possible and may have a certain effect on the progression of lung adenocarcinoma, which is supported by the results of the current study.

There are very few studies evaluating the distribution between the CA9 SNP and the EGFR phenotypes. In the current study, the ratio of CA9 SNP rs2071676 AG + GG was significantly lower in males with EGFR mutations of L858R expression. The L858R expression is a common type of EGFR mutation in lung adenocarcinoma, which related to a longer overall survival and lower tumor mutation burden value compared to EGFR wild type [45,46], but the prognosis of a L858R mutation is worse than the deletions in exon 19 phenotype [17,47,48]. As the polymorphism of CA9 indicates a relatively fair outcome of lung carcinoma, the lower ratio of CA9 SNP in lung adenocarcinoma with L858R expression may be considered as a co-predictor for the progression and prognosis but the detail needs further investigation. Regarding other CA9 SNP and EGFR phenotypes that revealed insignificant relationship, all the three CA9 SNP showed similar trends in different EGFR phenotype, such that the CA9 SNP rs2071676 and CA9 SNP rs3829078 had a numerically lower ratio in all the EGFR phenotypes compared to the CA9 wild type, while the expression of CA9 SNP rs1048638 was more frequent for all the EGFR phenotypes. Thus, the different CA9 SNPs may have a contrary expression with the different phenotypes of EGFR.

Concerning the clinicopathologic characteristics of lung adenocarcinoma and related EGFR phenotypes as well as CA9 SNP expression in the current study, the co-existence of CA9 SNP rs2071676 and EGFR wild type were significantly associated with both the lower tumor stage and lower risk for developing lymph node metastasis of lung adenocarcinoma whether in the whole study population or the male population. To our knowledge, this is a preliminary experience to demonstrate the distribution between CA9 SNP and EGFR phenotype that could affect the clinicopathologic characteristics of lung adenocarcinoma, which may lower the risk of disease deterioration [49,50]. To be more specific, the presence of CA9 SNP rs2071676 is correlated with a lower rate of advanced tumor stage in all types of EGFR, but became non-significant in lung adenocarcinoma patients with an EGFR mutation, implying the distribution of specific CA9 SNP and the phenotypes of EGFR may be similar if not identical. For the male population, the significantly lower tumor stage and lower risk for developing lymph node metastasis still presented in those with EGFR wild type and CA9 SNP rs2071676, and the tumor stage in all forms of EGFR with CA9 SNP rs2071676 showed non-significant results compared to those with wild type CA9. However, the percentage of early tumor stage was still numerically higher in lung adenocarcinoma males with CA9 SNP rs2071676, which might imply the CA9 SNP rs2071676 has a universal effect on the retardation of tumor progression. The other clinicopathological characteristics of lung adenocarcinoma involving tumor T-status, distal metastasis percentage, and cell differentiation grading did not show significant alteration under the presence of CA9 SNP rs2071676. Other genetic polymorphism, such as metastasis-associated lung adenocarcinoma transcript 1 that influence those characters [51], may be associated with the CA9 SNP distribution, although this needs further evaluation.

## 5. Conclusions

In conclusion, the appearance of CA9 SNP rs2071676 is negatively associated with the rate of EGFR L858R expression in the male population of lung adenocarcinoma. Furthermore, the CA9 SNP rs2071676 is significantly correlated to both a lower tumor stage and lower risk for developing lymph node metastasis, whereas in the whole population of the male subgroup of lung adenocarcinoma, these results are mainly accompanied with the EGFR wild type. The results of the current study may indicate that CA9 SNP rs2071676 could be used as a predictor of slow progression of lung adenocarcinoma. Further large cohort studies investigating the effect of CA9 SNP and EGFR phenotypes on the long-term survival rate of lung adenocarcinoma is mandatory.

## Figures and Tables

**Table 1:** Demographics and clinical characteristics of 474 patients in lung adenocarcinoma with epidermal growth factor receptor mutation status.

Variable	EGFR Wild Type (*N* = 193) *n* (%)	EGFR Mutation (*N* = 281) *n* (%)	*p*-Value
**Age**			
Mean ± SD	65.31 ± 13.46	64.83 ± 12.74	*p* = 0.689
<65	97 (50.3%)	143 (50.9%)	*p* = 0.893
≥65	96 (49.7%)	138 (49.1%)	
**Gender**			
Male	118 (61.1%)	95 (33.8%)	*p* < 0.001
Female	75 (38.9%)	186 (66.2%)	
**Cigarette smoking status**			
Never-smoker	88 (45.6%)	224 (79.7%)	*p* < 0.001
Ever-smoker	105 (54.4%)	57 (20.3%)	
**Stage**			
I + II	47 (24.4%)	73 (26.0%)	*p* = 0.689
III + IV	146 (75.6%)	208 (74.0%)	
**Tumor T-status**			
T1 + T2	100 (51.8%)	158 (56.2%)	*p* = 0.343
T3 + T4	93 (48.2%)	123 (43.8%)	
**Lymph node status**			
Negative	57 (29.5%)	83 (29.5%)	*p* = 0.999
Positive	136 (70.5%)	198 (70.5%)	
**Distant Metastasis**			
Negative	90 (46.6%)	119 (42.3%)	*p* = 0.356
Positive	103 (53.4%)	162 (57.7%)	
**Cell differentiation**			
Well	16 (8.3%)	31 (11.0%)	*p* < 0.001
Moderately	123 (63.7%)	221 (78.6%)	
Poorly	54 (28.0%)	29 (10.3%)	

EGFR: epidermal growth factor receptor; SD: standard deviation; N: number.

**Table 2:** Distribution frequency of carbonic anhydrase 9 genotypes of patients with lung adenocarcinoma and multiple logistic regression analysis of epidermal growth factor receptor mutation association.

SNP Genotypes	Wild Type (*N* = 193)	EGFR Mutation (All)		L858R		Exon 19 in-Frame Deletion	
		(*N* = 281)	AOR ^#^ (95% CI)	(*N* = 141)	AOR ^#^ (95% CI)	(*N* = 122)	AOR ^#^ (95% CI)
**rs2071676**							
AA	37 (19.2%)	65 (23.1%)	1.00	31 (22.0%)	1.00	30 (24.6%)	1.00
AG	115 (59.6%)	147 (52.3%)	0.82 (0.50–1.36)	68 (48.2%)	0.76 (0.41–1.42)	67 (54.9%)	0.87 (0.48–1.59)
GG	41 (21.2%)	69 (24.6%)	0.97 (0.53–1.77)	42 (29.8%)	1.26 (0.62–2.58)	25 (20.5%)	0.79 (0.38–1.64)
AG + GG	156 (80.8%)	216 (76.9%)	0.86 (0.53–1.40)	110 (78.0%)	0.89 (0.50–1.61)	92 (75.4%)	0.85 (0.47–1.51)
**rs3829078**							
AA	172 (89.1%)	258 (91.8%)	1.00	125 (88.7%)	1.00	115 (94.3%)	1.00
AG	21 (10.9%)	23 (8.2%)	0.70 (0.36–1.35)	16 (11.3%)	0.88 (0.41–1.86)	7 (5.7%)	0.52 (0.21–1.31)
GG	0 (0 %)	0 (0 %)	---	0 (0 %)	---	0 (0 %)	---
AG + GG	21 (10.9%)	23 (8.2%)	0.70 (0.36–1.35)	16 (11.3%)	0.88 (0.41–1.86)	7 (5.7%)	0.52 (0.21–1.31)
**rs1048638**							
CC	173 (89.6%)	239 (85.1%)	1.00	122 (86.5%)	1.00	102 (83.6%)	1.00
CA	20 (10.4%)	37 (13.2%)	1.61 (0.86–3.01)	17 (12.1%)	1.58 (0.72–3.45)	17 (13.9%)	1.84 (0.88–3.87)
AA	0 (0.0%)	5 (1.7%)	---	2 (1.4%)	---	3 (2.5%)	---
CA + AA	20 (10.4%)	42 (14.9%)	1.76 (0.96–3.25)	19 (13.5%)	1.74 (0.82–3.73)	20 (16.4%)	2.03 (0.99–4.15)

SNP: single nucleotide polymorphism; EGFR: epidermal growth factor receptor; N: number; AOR: adjusted odds ratio; CI: confidence interval; **^#^** The AORs with 95% CIs were estimated by multiple logistic regression models after controlling for age, gender and cigarette smoking status.

**Table 3:** Distribution frequency of carbonic anhydrase 9 genotypes of 213 male patients with lung adenocarcinoma and multiple logistic regression analysis of epidermal growth factor receptor mutation association.

SNP Genotypes	Wild Type (*N* = 118)	EGFR Mutation (All)		L858R		Exon 19 in-Frame Deletion	
		(*N* = 95)	AOR ^#^ (95% CI)	(*N* = 37)	AOR ^#^ (95% CI)	(*N* = 49)	AOR ^#^ (95% CI)
**rs2071676**							
AA	20 (16.9%)	26 (27.4%)	1.00	12 (32.4%)	1.00	13 (26.5%)	1.00
AG	76 (64.4%)	54 (56.8%)	0.61 (0.30–1.24)	18 (48.6%)	0.40 (0.16–0.95) *^,a^	30 (61.2%)	0.69 (0.29–1.63)
GG	22 (18.7%)	15 (15.8%)	0.68 (0.27–1.71)	7 (19.0%)	0.53 (0.17–1.62)	6 (12.3%)	0.51 (0.15–1.68)
AG + GG	98 (83.1%)	69 (72.6%)	0.63 (0.32–1.25)	25 (67.6%)	0.43 (0.18–0.98) *^,b^	36 (73.5%)	0.65 (0.28–1.51)
**rs3829078**							
AA	105 (89.0%)	89 (93.7%)	1.00	35 (94.6%)	1.00	45 (91.8%)	1.00
AG	13 (11.0%)	6 (6.3%)	0.58 (0.20–1.63)	2 (5.4%)	0.43 (0.09–2.10)	4 (8.2%)	0.80 (0.24–2.68)
GG	0 (0 %)	0 (0 %)	---	0 (0 %)	---	0 (0 %)	---
AG + GG	13 (11.0%)	6 (6.3%)	0.58 (0.20–1.63)	2 (5.4%)	0.43 (0.09–2.10)	4 (8.2%)	0.80 (0.24–2.68)
**rs1048638**							
CC	101 (85.6%)	77 (81.1%)	1.00	30 (81.1%)	1.00	39 (79.6%)	1.00
CA	17 (14.4%)	16 (16.8%)	1.17 (0.54–2.52)	6 (16.2%)	1.02 (0.35–2.94)	9 (18.4%)	1.39 (0.55–3.53)
AA	0 (0.0%)	2 (2.1%)	---	1 (2.7%)	---	1 (2.0%)	---
CA + AA	17 (14.4%)	18 (18.9%)	1.27 (0.60–2.70)	7 (18.9%)	1.16 (0.42–3.21)	10 (20.4%)	1.49 (0.60–3.70)

SNP: single nucleotide polymorphism; EGFR: epidermal growth factor receptor; N: number; AOR: adjusted odds ratio; CI: confidence interval; **^#^** The AORs with 95% CIs were estimated by multiple logistic regression models after controlling for age, gender and cigarette smoking status. * denotes significant difference of the carbonic anhydrase 9 polymorphism distribution compared to the wild type. ^a^ *p* = 0.039; ^b^ *p* = 0.046.

**Table 4:** Clinicopathologic characteristics of lung adenocarcinoma patients with epidermal growth factor receptor mutation, stratified by polymorphic genotypes of carbonic anhydrase 9 rs2071676.

Variable	ALL (*N* = 474)			EGFR Wild Type (*N* = 193)			EGFR Mutation (*N* = 281)		
	AA (*N* = 102)	AG + GG (*N* = 372)	*p*-Value	AA (*N* = 37)	AG + GG (*N* = 156)	*p*-Value	AA (*N* = 65)	AG + GG (*N* = 216)	*p*-Value
**Stages**									
I + II	18 (17.6%)	102 (27.4%)	*p* = 0.044 *	4 (10.8%)	43 (27.6%)	*p* = 0.033 *	14 (21.5%)	59 (27.3%)	*p* = 0.352
III + IV	84 (82.4%)	270 (72.6%)		33 (89.2%)	113 (72.4%)		51 (78.5%)	157 (72.7%)	
**Tumor T status**									
T1 + T2	60 (58.8%)	198 (53.2%)	*p* = 0.315	22 (59.5%)	78 (50.0%)	*p* = 0.301	38 (58.5%)	120 (55.6%)	*p* = 0.679
T3 + T4	42 (41.2%)	174 (46.8%)		15 (40.5%)	78 (50.0%)		27 (41.5%)	96 (44.4%)	
**Lymph node status**									
Negative	27 (26.5%)	113 (30.4%)	*p* = 0.444	4 (10.8%)	53 (34.0%)	*p* = 0.005 *	23 (35.4%)	60 (27.8%)	*p* = 0.239
Positive	75 (73.5%)	259 (69.6%)		33 (89.2%)	103 (66.0%)		42 (64.6%)	156 (72.7%)	
**Distant metastasis**									
Negative	45 (44.1%)	164 (44.1%)	*p* = 0.995	15 (40.5%)	75 (48.1%)	*p* = 0.409	30 (46.2%)	89 (41.2%)	*p* = 0.479
Positive	57 (55.9%)	208 (55.9%)		22 (59.5%)	81 (51.9%)		35 (53.8%)	127 (58.8%)	
**Cell differentiation**									
Well/Moderately	87 (85.3%)	304 (81.7%)	*p* = 0.400	28 (75.7%)	111 (71.2%)	*p* = 0.582	59 (90.8%)	193 (89.4%)	*p* = 0.742
Poorly	15 (14.7%)	68 (18.3%)		9 (24.3%)	45 (28.8%)		6 (9.2%)	23 (10.6%)	

EGFR: epidermal growth factor receptor; N: number; * denotes significant difference about the distribution of the clinicopathologic characteristics of lung adenocarcinoma.

**Table 5:** Clinicopathologic characteristics of 213 male lung adenocarcinoma patients with epidermal growth factor receptor mutation, stratified by polymorphic genotypes of carbonic anhydrase 9 rs2071676.

Variable	ALL (*N* = 213)			EGFR Wild Type (*N* = 118)			EGFR Mutation (*N* = 95)		
	AA (*N* = 46)	AG + GG (*N* = 167)	*p*-Value	AA (*N* = 20)	AG + GG (*N* = 98)	*p*-Value	AA (*N* = 26)	AG + GG (*N* = 69)	*p*-Value
**Stages**									
I + II	7 (15.2%)	42 (25.1%)	*p* = 0.156	1 (5.0%)	26 (26.5%)	*p* = 0.037 *	6 (23.1%)	16 (23.2%)	*p* = 0.991
III + IV	39 (84.8%)	125 (74.9%)		19 (95.0%)	72 (73.5%)		20 (76.9%)	53 (76.8%)	
**Tumor T-status**									
T1 + T2	27 (58.7%)	86 (51.5%)	*p* = 0.386	11 (55.0%)	50 (51.0%)	*p* = 0.746	16 (61.5%)	36 (52.2%)	*p* = 0.414
T3 + T4	19 (41.3%)	81 (48.5%)		9 (45.0%)	48 (49.0%)		10 (38.5%)	33 (47.8%)	
**Lymph node status**									
Negative	8 (17.4%)	47 (28.1%)	*p* = 0.140	0 (0%)	31 (31.6%)	*p* = 0.003 *	8 (30.8%)	16 (23.2%)	*p* = 0.448
Positive	38 (82.6%)	120 (71.9%)		20 (100%)	67 (68.4%)		18 (69.2%)	53 (76.8%)	
**Distant metastasis**									
Negative	19 (41.3%)	77 (46.1%)	*p* = 0.562	7 (35.0%)	50 (51.0%)	*p* = 0.191	12 (46.2%)	27 (39.1%)	*p* = 0.535
Positive	27 (58.7%)	90 (53.9%)		13 (65.0%)	48 (49.0%)		14 (53.8%)	42 (60.9%)	
**Cell differentiation**									
Well/Moderately	39 (84.8%)	123 (73.7%)	*p* = 0.117	16 (80.0%)	64 (65.3%)	*p* = 0.200	23 (88.5%)	59 (85.5%)	*p* = 0.709
Poorly	7 (15.2%)	44 (26.3%)		4 (20.0%)	34 (34.7%)		3 (11.5%)	10 (14.5%)	

EGFR: epidermal growth factor receptor; N: number; * denotes significant difference about the distribution of the clinicopathologic characteristics of lung adenocarcinoma.
